# Role of *APOBEC3F* Gene Variation in HIV-1 Disease Progression and Pneumocystis Pneumonia

**DOI:** 10.1371/journal.pgen.1005921

**Published:** 2016-03-04

**Authors:** Ping An, Sudhir Penugonda, Christian W. Thorball, Istvan Bartha, James J. Goedert, Sharyne Donfield, Susan Buchbinder, Elizabeth Binns-Roemer, Gregory D. Kirk, Wenyan Zhang, Jacques Fellay, Xiao-Fang Yu, Cheryl A. Winkler

**Affiliations:** 1 Basic Research Laboratory, Center for Cancer Research, National Cancer Institute, Leidos Biomedical Research, Inc., Frederick National Laboratory for Cancer Research, Frederick, Maryland, United States of America; 2 Department of Medicine, Northwestern University Feinberg School of Medicine, Chicago, Illinois, United States of America; 3 School of Life Sciences, École Polytechnique Fédérale de Lausanne, Lausanne, Switzerland; 4 Swiss Institute of Bioinformatics, Lausanne, Switzerland; 5 Infections and Immunoepidemiology Branch, Division of Cancer Epidemiology and Genetics, National Cancer Institute, Bethesda, Maryland, United States of America; 6 Rho, Inc., Chapel Hill, North Carolina, United States of America; 7 San Francisco Department of Public Health, San Francisco, California, United States of America; 8 Department of Epidemiology, Johns Hopkins School of Public Health, Baltimore, Maryland, United States of America; 9 Institute of Virology and AIDS Research, First Hospital of Jilin University, Changchun, China; 10 Department of Molecular Microbiology and Immunology, Johns Hopkins School of Public Health, Baltimore, Maryland, United States of America; Ospedale San Pietro Fatebenefratelli, ITALY

## Abstract

Human APOBEC3 cytidine deaminases are intrinsic resistance factors to HIV-1. However, HIV-1 encodes a viral infectivity factor (Vif) that degrades APOBEC3 proteins. *In vitro* APOBEC3F (A3F) anti-HIV-1 activity is weaker than A3G but is partially resistant to Vif degradation unlike A3G. It is unknown whether A3F protein affects HIV-1 disease *in vivo*. To assess the effect of *A3F* gene on host susceptibility to HIV- acquisition and disease progression, we performed a genetic association study in six well-characterized HIV-1 natural cohorts. A common six-Single Nucleotide Polymorphism (SNP) haplotype of *A3F* tagged by a codon-changing variant (p. I231V, with allele (V) frequency of 48% in European Americans) was associated with significantly lower set-point viral load and slower rate of progression to AIDS (Relative Hazards (RH) = 0.71, 95% CI: 0.56, 0.91) and delayed development of pneumocystis pneumonia (PCP) (RH = 0.53, 95% CI: 0.37–0.76). A validation study in the International Collaboration for the Genomics of HIV (ICGH) showed a consistent association with lower set-point viral load. An in vitro assay revealed that the *A3F* I231V variant may influence Vif mediated A3F degradation. Our results provide genetic epidemiological evidence that A3F modulates HIV-1/AIDS disease progression.

## Author Summary

Cytidine deaminases of the human APOBEC3 (*A3*) gene family serve as intrinsic resistance factors to HIV-1 and other retroviruses. HIV-1 encodes the viral infectivity factor (Vif) protein that degrades APOBEC3 proteins via the ubiquitination-proteosomal pathway. APOBEC3F (A3F), unlike APOBEC3G (A3G), is partially resistant to Vif-mediated degradation. The antiviral activity of the A3 family has largely been demonstrated in *in vitro* experiments, and there is mounting evidence that *A3G* genetic variants influence HIV disease progression. It is not resolved if A3F protein affects HIV disease *in vivo*. To assess the *in vivo* effect of A3F, we performed a genetic association study of genetic variants in *A3F* for their influence on HIV- acquisition and HIV disease progression. A common *A3F* haplotype was associated with a 30% reduced rate of AIDS disease progression, lower set-point viral load and delayed development of pneumocystis pneumonia (PCP) in European Americans. This study provides the first epidemiological evidence that A3F might modify HIV-1/AIDS pathogenesis.

## Introduction

The apolipoprotein B mRNA-editing enzyme catalytic polypeptide-like 3 (APOBEC3, A3) proteins are a family of cellular cytidine deaminases that defend against a diverse set of retroviruses, endogenous retroelements and DNA viruses, including human immunodeficiency virus type I (HIV-1) [1–5]. Humans A3 proteins are encoded by seven *A3* genes (*A3A*, *A3B*, *A3C*, *A3D*, *A3F*, *A3G*, and *A3H*) tandemly arrayed on chromosome 22. In *in vitro* experimental systems, several members of the human APOBEC3 family are capable of inhibiting HIV-1 replication to some degree (A3G, A3F, A3D and some A3H haplotypes), with A3G and A3F showing evidence of strong inhibitory activity. A3G protein catalyzes deamination of cytosine bases on the DNA minus strand during reverse transcription, inducing guanosine (G)-to-adenosine (A) hypermutation in the HIV-1 provirus [2,6–8]. A3G causes GG-to-AG transitions, while APOBEC3F causes GA-to-AA nucleotide changes. However, HIV-1 encodes an accessory protein, viral infectivity factor (Vif), to counteract APOBEC3 proteins by mediating the proteasomal degradation of A3 proteins [9–13]. It is unknown whether the anti-HIV-1 activity of A3 proteins is completely neutralized by Vif or if A3 still exerts meaningful antiviral effect in vivo. While G to A hypermutation of the HIV genome is deleterious to the virus [10], [16–18], a recent in vitro study showed sub-lethal levels of A3G induced G to A mutations may contribute to viral diversity, which consequently may contribute to immune escape and drug resistance [14]. How A3 proteins’ opposing antiviral and viral mutation properties contribute to HIV-1 pathogenesis in vivo is a critical question.

The antiviral strength of A3F is unresolved by *in vitro* experiments as some reported that A3F activity is as strong as A3G [7,15–17] while others indicated that it is weaker [18–21], possibly due to varied experimental settings. A3F is highly expressed in CD4+ T-cells, the cells infected by HIV-1 [22,23]. Unlike A3G, A3F is partially resistant to Vif-mediated degradation [15]. A3F also has a favored deamination sequence target that differs from A3G (5′-TC for A3F and 5′-CC for A3G) [24]. Patterns of HIV DNA hypermutation observed in HIV-1 infected individuals are consistent with the notion that both A3G and A3F might be acting on HIV-1 *in vivo* [25].

Testing the *in vivo* role of intrinsic host restriction factors such as A3 proteins are challenging due to a lack of a good animal model [26,27]. The genetic associations between natural polymorphisms in *A3* genes and resistance to infection to HIV-1or restriction to HIV-1 disease progression would provide critical evidence supporting an *in vivo* role for A3 proteins in restricting HIV. Genetic variation in *A3G* and *A3H* have been shown to associate with HIV-1 disease progression [28–30], supporting the *in vivo* activity of A3G and A3H’s. However, it remains unknown whether A3F confers any appreciable effect on HIV acquisition or HIV pathogenesis. We investigated the association of variants in the *A3F* gene with susceptibility to HIV-1 acquisition, HIV-1 viral load, and progression to AIDS in six clinically well-characterized, natural history HIV cohorts.

## Results

### Linkage disequilibrium, haplotype structure and characteristics of *A3F* variants

**Fig 1:**
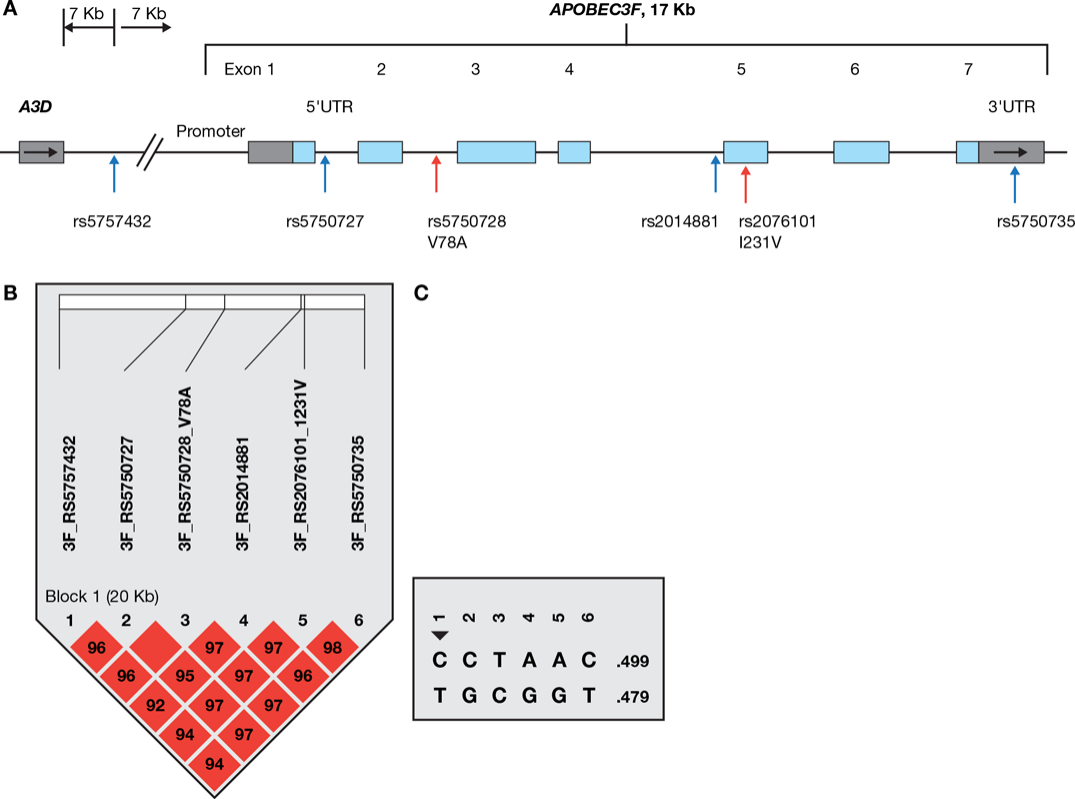
SNPs analyzed in the *A3F* gene. (A) Gene structure and SNP locations. The colored blocks indicate exons, empty blocks, untranslated regions (UTR), horizontal arrows, the direction of transcription and vertical arrows, the positions of SNPs. (B) Linkage disequilibrium matrix of SNPs in the *A3F* gene region in European Americans, as illustrated by Haploview [74]. Red block indicates D’ = 1.0, and the number in the blocks indicates the value of D’. The linkage disequilibrium block depicted by black triangle was based on the 95% Confidence interval criteria. (C) Haplotypes in the *A3F* gene region in European Americans.

*A3F* is about 17 kb in length, containing 7 exons. Six potentially functional SNPs, including the missense SNP rs2076101 ATC>GTC, coding p.I231V, were genotyped in the AIDS cohorts (**Fig 1** and **Table 1**). The allele rs2076101G (231V) had a frequency of 48% in European Americans (EA) and 76% in African Americans (AA) in our cohorts. Genotype distributions of the 6 SNPs conformed to Hardy-Weinberg equilibrium expectations (*P* > 0.05) in European Americans and African Americans. The 6 SNPs were in near-absolute linkage disequilibrium (LD) and highly correlated (D’ = 1 and *r*^2^>0.95, respectively) in EA. SNP rs2076101A/G (p.I231V) tags the most frequent A3F haplotype comprising the variant alleles of all 6 SNPs (**Fig 1**); we therefore used rs2076101A/G (p.I231V) to represent the *A3F* haplotype in the association analyses.

Based on HapMap LD map covering the APOBEC3 gene family region (**S1 Fig**), *A3F*, *3G*, *3H* each gene forms a distinct haplotype block but are not in strong LD with each other, as reported previously [29].

**Table 1:** Characteristics of *A3F* genetic variants.

SNP rs#	Position on Chr. 22	Location	codon change	Allele change	Predicted SNP function^a^	Polyphen^b^
rs5757432	37758865	Intergenic		C/T	miRNA	
rs5750727	37767356	Intron		C/G	TFBS	
rs5750728	37770095	Intron or alternative exon	V78A	C/T	TFBS Splicing(ESE)	benign
rs2014881	37775326	Intron		A/G		
rs2076101	37775500	exon	I231V	A/G	Splicing(ESE), nsSNP	possibly damaging
rs5750735	37779601	3'UTR		C/T	miRNA	

^a^Predicated by SNPinfo web server [http://snpinfo.niehs.nih.gov]); TFBS, transcription factor-binding site; miRNA, MicroRNA-binding sites; nsSNP, non-synonymous SNP; ESE, exonic splicing enhancer.

^b^Predicated by Variant Effect Predictor from Ensembl (www.ensembl.org/)

The possible functional consequence of the six SNPs, as evaluated by the Variant Effect Predictor (VEP) function from Ensembl (www.ensembl.org/), is listed in **Table 1**. The missense SNP rs2076101 (p.I231V) results in a change from isoleucine to valine, has a Polyphen score of 0.90, reflecting a non-conservative change with possible deleterious consequence. SNP rs5750728 is located in the intron of A3F encoding the major transcript; however, the shorter transcript encoding 101 amino acids (NM_0010066666) would contain p.V78A, a benign change. Whether this isoform is functional *in vivo* is still unknown. SNP rs5750727 in intron 1 is located within an experimentally identified regulatory feature in CD4+ T-cells (ENSR00001532770, based on Ensembl database).

### Impact of *A3F* 231V haplotype on HIV-1 progression

**Fig 2:**
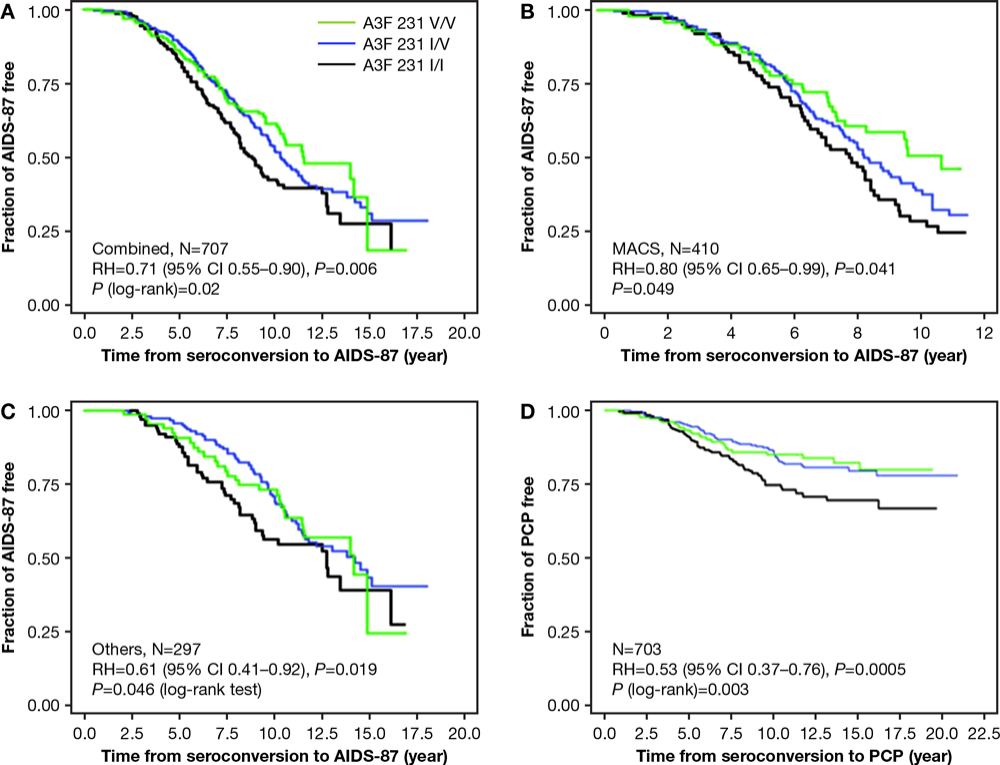
Genetic effect of *A3F* 231V haplotype on AIDS progression. Kaplan-Meier survival curves of *A3F*-231I/V genotype carriers progression to AIDS since HIV-1 infection in the (A) combined cohorts. (B) MACS cohort. (C) other cohorts. (D). Kaplan-Meier survival curves of *A3F*-231I/V genotype carriers for progression to PCP since HIV-1 infection in 703 seroconverter European Americans. RH and adjusted *P* values were obtained from a Cox proportional hazards model. *P* values for survival curves were obtained from a log-rank test.

To assess the impact of the *A3F* 231V haplotype on disease progression from the estimated date of seroconversion to clinical AIDS, we performed time-to-event analysis for 707 European American seroconverters. The seroconversion date was estimated as the midpoint between the last seronegative and the first seropositive HIV-1 antibody test date (mean seroconversion interval 0.89 years, range 0.06–3.0 years). In the Kaplan-Meier survival curve analysis, p. 231V was associated with delayed progression to clinical AIDS (**Fig 2A**) (*P* = 0.02, in both log-rank and Wilcoxon test) in the dominant genetic model. To account for covariates (age, sex, and cohort) and genetic confounders (*CCR5* and *HLA* variants), we employed adjusted Cox proportional hazards regression models to evaluate progression rates to clinical AIDS in 707 European American seroconverters. After stratifying by age group, sex and cohort, carriers of one or two copies of p. 231V showed significantly delayed progression to clinical AIDS (dominant genetic model, RH = 0.68, 95% CI, 0.54–0.87, *P* = 0.002); In the model adjusted for the known genetic factors of *HLA* alleles (*HLA-C*, *B27*, *B57*, *B35Px*, *HLA* class I homozygosity, and *CCR5* (p1 and Δ32)[31,32], the association remained significant (RH = 0.71, 95% CI, 0.56–0.91, *P*_adj._ = 0.007, **Table 2**). Further adjustment for potential population stratification using the first 2 eigenvalues led to similar result (RH = 0.71, 95% CI, 0.55–0. 90; *P*_*eigen*_ = 0.006). The *A3F* association was not attenuated (RH = 0.68, 95% CI, 0.51–0. 90) with additional adjustment for the known HIV progression modifiers *A3B* gene deletion [33] and *A3G* 199376C [30]. The effect was not affected by the potential seroconversion estimate errors; RHs were changed from 0.71 to 0.72, 0.72, respectively, when seroconversion intervals were shortened to < 2.0 (n = 649) or to <1.5 years (n = 585). The additive genetic model also showed significant association (RH = 0.80, 95% CI, 0.68–0.95, *P*_*eigen*_ = 0.009). Bayesian analysis using the Gibbs sampler with 50,000 iterations provided the same results, indicating the p values are robust. Separated analyses conducted in the MACS cohort alone (**Fig 2B**) or in the other cohorts (**Fig 2C**) showed significant association in the same direction. These results provide evidence that the haplotype carrying the 6 variant alleles including p. 231V was associated with a 30% reduced rate of AIDS disease in European Americans. In 281 African Americans seroconverters, p. 231V trended in the same direction in a Cox model analysis (RH = 0.35, 95% CI, 0.10–1.19, *P*_adj._ = 0.09; RH = 0.30, 95% CI, 0.07–1.37, *P* = 0.12 with further adjustment of population stratification).

**Table 2:** Impact of *A3F* 231V haplotype on AIDS progression assessed by Cox proportional hazards regression model.

Population	Disease outcome	no. total/events	Adjustment	RH (95% CI)	*P*
European American	AIDS 1987	707/318	unadjusted	0.68 (0.54–0.87)	0.002
		707/318	adjusted^a^	0.71 (0.55–0.90)	0.006
	PCP	703/134	unadjusted	0.53 (0.37–0.76)	0.0005
Africa American	AIDS 1987	281/48	unadjusted	0.39 (0.12–1.31)	0.12
		281/48	adjusted^b^	0.34 (0.10–1.18)	0.09

Dominant genetic model (231V/V or V/I vs. I/I) was tested and was stratified by sex, age and cohort.

^a^ adjusted for covariates *HLA* homozygosity, *HLA-C*, *HLA*-B*57, *HLA*-B*35PX, *HLA*-B*27, *CCR5*-**Δ**32, *CCR5*-59029 and the first 2 eigenvalues

^b^ adjusted for covariates *HLA* homozygosity, HLA-B*57, *HLA*-B*35PX, HLA-B*27, and *CCR5*-59029.

The protective effect of p. 231V for AIDS progression was also observed in a defined disease category analysis (**S2 Fig**), which allows the addition of seroprevalent patients who had not progressed to AIDS for >10 years (time from seroconversion to AIDS) to the slow progressor category of seroconverters; only seroconverters were included in the fast category (≤10 years) to avoid frailty bias. The prevalence of AIDS protective genotypes 231V/V and 231 I/V was elevated in the slow progressors (Odds ratio (OR) = 0.62, *P* = 0.004, *chi*^2^ test, **S2 Fig**). The results of both the survival (**Fig 2A** and **Table 2**) and defined disease category analyses (**S2 Fig**) support a strong dominant 231V association with delayed progression to clinical AIDS outcome.

### *A3F* 231V haplotype association with pneumocystis pneumonia (PCP)

Multiple different AIDS-defining conditions including opportunistic infections and malignancies are AIDS-defining [34]. In an explanatory data analysis, we tested whether the common AIDS defining condition, pneumocystis pneumonia was influenced by p.231V. In a Kaplan-Meier survival curve analysis of 703 seroconverters, carriers of 1 or 2 copies of the p. 231V haplotype showed a significantly slower progression to pneumocystis pneumonia, compared with 231I/I carriers (log-rank test, *P* = 0.003; Wilcoxon test, *P* = 0.005, **Fig 2D**). A Cox model analysis also showed an association of p.231V with delayed progression to PCP (dominant model, RH = 0.53, 95% CI 0.37–0.76, Wald test *P*_*dom*_ = 0.0005, **Table 2**; additive model, RH = 0.69, 95% CI 0.54–0.89, *P* = 0.004; both adjusted for age, sex and cohort). A sensitivity test in the MACS cohort revealed no impact of PCP prophylaxis usage on the association. In a sensitivity test performed in 410 MACS seroconverters, adjustment of PCP prophylaxis (n = 138) did not attenuate the association of p.231V with PCP (RH changed from 0.637 to 0.633). These results demonstrated that p.231V has a protective association with PCP development. In 278 Africa American seroconverters, a nonsignificant protective trend to PCP was seen for p.231V homozygote carriers (OR = 0.55, 95% CI, 0.28–1.12, *P* = 0.10; **S3 Fig**)

### *A3F* 231V haplotype association with HIV-1 viral load

Among 183 MACS seroconverters with available viral load set-point data, individuals carrying the p.231V (n = 141) had significantly lower mean viral load than those homozygous for the p.231I (n = 42) (mean 4.46 ± 0.69 versus 4.72 ± 0.46 log10 copies/ml; *P* = 0.02). The p.231V carriers were associated with a 0.27 ± 0.65 log_10_ lower set-point viral load (equivalent of 1.86-fold copies per ml of plasma). For comparison, heterozygousity for *CCR5* Δ32 [35], had a 0.23 ± 0.65 lower viral load (*P* = 0.04) in this dataset, supporting the validity of the analysis.

**Fig 3:**
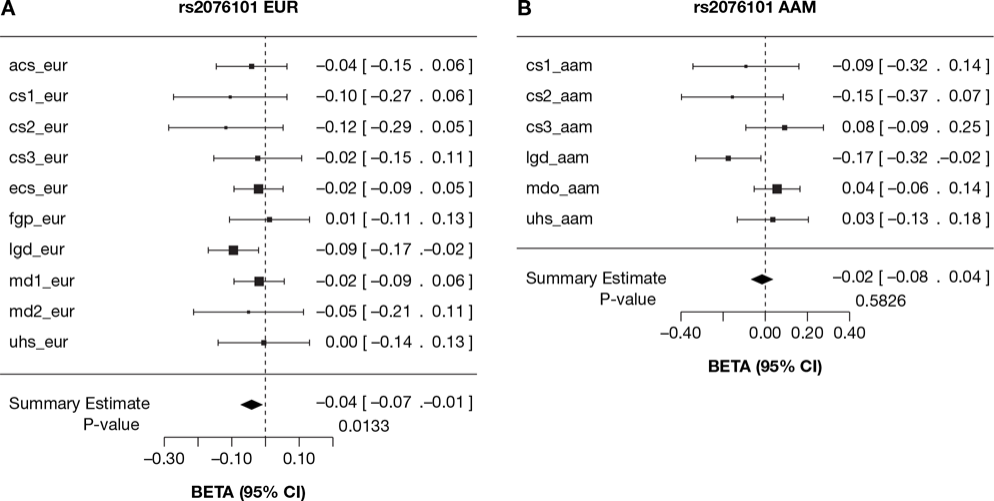
HIV-1 viral load levels of *A3F* 231V allele carriers in multiple HIV-1 cohorts. Forest plot of effect estimates of HIV-1 viral load for the *A3F* rs2076101 G allele with 95% confidence intervals per study group (box and whiskers) and after meta-analysis (diamond) in the International Collaboration for the Genomics of HIV (ICGH) consortium [36]. (A) Meta-analyses for European or European-descents (eur). (B) Meta-analyses for African Americans (aam). A description of the study groups and cohorts used in the meta-analysis is presented in S3 Table.

To replicate the viral load association result, we obtained the association results of the *A3F* SNPs with HIV-1 viral load from the International Collaboration for the Genomics of HIV (ICGH) that combined genome-wide SNP data from 25 cohorts worldwide [36]. We analyzed 13 Europe- or USA-based cohorts in ICGH including 6 cohorts used in this study with viral load data available. For independent confirmation, we removed 183 MACS seroconverter subjects used in the above viral load analysis from the ICGH. A meta-analysis of 13 cohorts comprising 10,395 seropositives showed a consistent association of *A3F* rs2076101 (231V) with lower set-point viral load (*β* = -0.04, 95% CI, -0.07, -0.01, *P* = 0.01) in EA (**Fig 3A**). The association was mainly driven by the cohorts used in this study (comprising cohorts MACS, MHCS, DCG, HGDS, SFCCC and ALIVE), and most other European-ancestry cohorts showed nonsignificant trends in the same direction. In African American subjects, a meta-analysis showed no association with viral load but a significant protective association was seen for the ALIVE cohort (95% CI of *β*, -0.32, -0.02, **Fig 3B**). Together, these results indicate a modest but consistent association between p.231V and lower viral load.

### Impact of *A3F* 231V on HIV-1 acquisition

No differences in the *A3F* p.231V frequencies were observed between African- or European American HIV-1 seronegative groups and HIV-1-infected group (**S1 Table**), indicating that the *A3F* genetic variation assessed herein has no obvious effect on susceptibility to HIV-1 acquisition.

### *A3F* rs5750728 alters transcriptional factor binding

**Fig 4:**
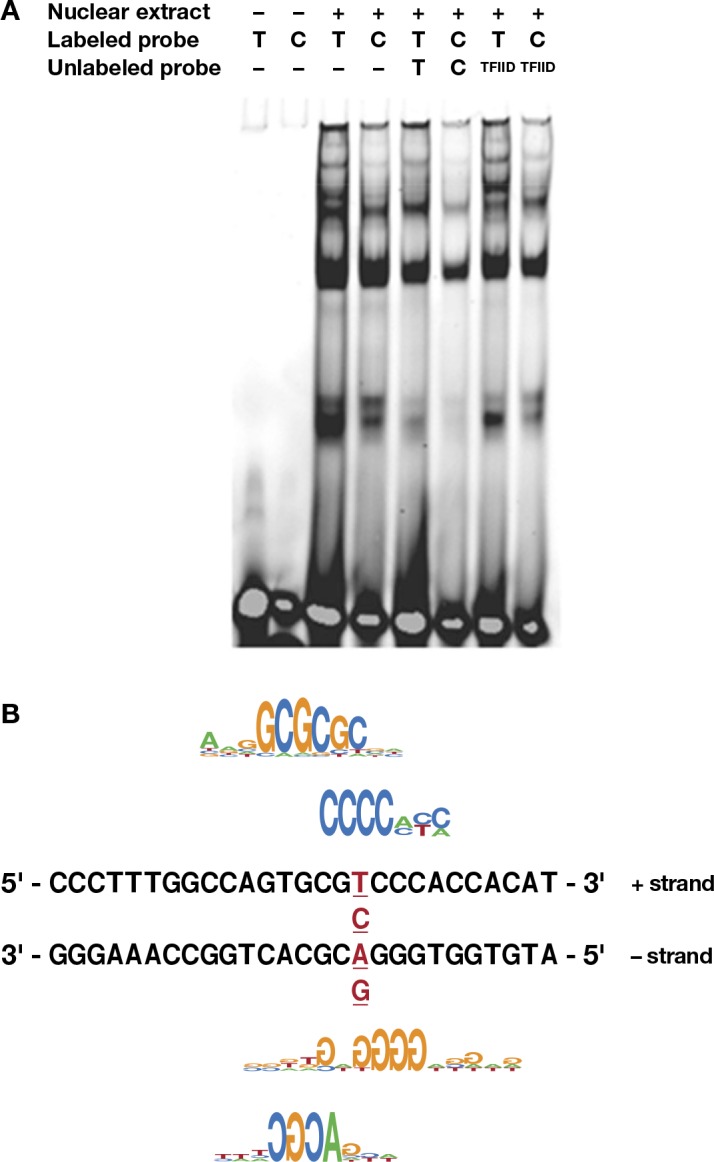
Allelic-specific protein binding of *A3F* rs5750728T/C. (A) For the EMSA, two *A3F* DNA fragments with rs5750728 T or C allele were incubated with HeLa nuclear extract. The specificity of the intense bands present in the rs5750728 T lane (lane 3) was demonstrated by adding 200-fold excess of unlabeled T oligo (lane 5) and unrelated TFIID oligos (lane 7) as competitors. (B) Transcription factor binding sites affected by the rs5750728 T/C change based on SNPInspector. The C allele of rs5750728 generates E2FF, ZKSCAN3 (matching the positive (+) strand of the rs5750728 residing *A3F* DNA fragment), KLFS and loss of WHNF (matching the negative—strand) sites. The rs5750728 site is in red and underlined.

The intronic SNP rs5750728 is in near absolute LD with 231V and is predicted to have regulatory function (**Table 1**). We used electrophoretic mobility shift assays (EMSA) to determine if the variant alters transcription factor binding. The rs5750728 T containing oligonucleotide probe formed a major DNA-protein complex with HeLa cell nuclear extract, which was nearly absent when using rs5750728 C containing oligonucleotide (**Fig 4A**, lanes 3 and 4). The DNA-protein complex was abolished by the addition of excessive unlabeled competitor probes (**Fig 4A,** lanes 5 and 6), but was not affected by non-specific competitor probes (**Fig 4A,** lanes 7 and 8), demonstrating the binding specificity. These results indicate that the rs5750728 C variant allele caused a loss of binding to certain transcription factors, although the identity of the transcription factors is not yet known.

Genomatix software SNPInspector predicted that rs5750728 T/C change generates transcription factor binding sites for E2FF (myc activator/cell cycle regulator), KLFS (Krueppel like transcription factors), and ZKSCAN3 (zinc finger with KRAB and SCAN domains 3), and loss of WHNF (winged helix binding sites) (**Fig 4B**). Another SNP rs5750727 C/G causes lost site for MRF2 (modulator recognition factor 2).

### Influence of *A3F* 231V haplotype on HIV-1 Vif mediated degradation

**Fig 5:**
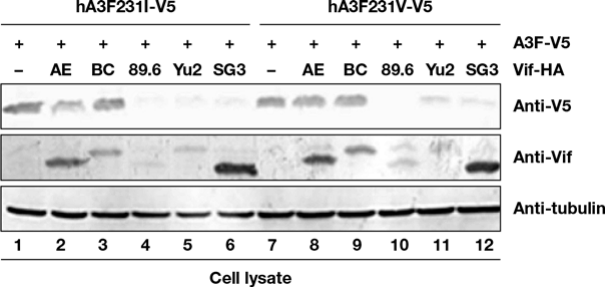
Influence of HIV-1 Vif on A3F variant protein expression. HEK293T cells in 12-well plate were co-transfected with 1 μg of Vif expression vector or a control vector, plus 0.3 μg of A3F-231I-V5 expression vector encoding a V5-tagged A3F231I protein (lanes 1–6), or plus 0.3 μg of A3F231V-V5 expression vector encoding a V5-tagged A3F-231V protein (lanes 7–12). At 48 h after transfection, cell lysates were harvested for immunoblot analysis with indicated antibodies.

HIV-1 Vif recruits Cul5-based CRL E3 ligase to target A3F for ubiquitination and degradation [37]. To determine whether A3F variants could alter A3F sensitivity to Vif mediated degradation, A3F I231-V5 and A3F V231-V5 expression vectors were co-transfected with several HIV-1 Vif expression vectors in HEK293T cells. A3F expression was determined by immunoblotting using anti-V5 antibodies. A3F I231-V5 and A3F V231-V5 were sensitive to strains 89.6, Yu2, and SG3 HIV-1 Vif mediated degradation but resistant to AE and BC HIV-1 Vif mediated degradation (Fig 5). Nevertheless, A3F V231-V5 was approximately 39% more resistant (based on normalized intensity) to HIV-1 AE Vif mediated degradation (Fig 5, lane 8) compared to A3F I231-V5 (Fig 5, lane 2). A3F V231-V5 was 30% more resistant to HIV-1 Yu2 Vif (Fig 5, lane 11) mediated degradation compared to A3F I231-V5 (Fig 5, lane 5). These data suggest that A3F variations at position 231 may influence the sensitivity of A3F to certain HIV-1 Vif mediated inactivation.

### Influence of *A3F* 231V haplotype on HIV-1 hypermutation

The effect of rs2076101 on the level of HIV hypermutation was tested using paired human genotypes and viral sequences from 421 patients in the Swiss HIV Cohort Study. Counts of GA-to-AA hypermutations likely induced by A3F were quantified as in the Hypermut2 tool [38]. We observed 8.11(out of 144.95 potential A3F specific G->A mutations, 5.5% hypermutation rate), 8.23 (5.6%) and 8.63 (5.3%) A3F specific G->A mutations in three rs2076101 genotype groups, respectively; the log odds ratio of hypermutation was not higher in individuals carrying the A3F 231V than those who don’t (coefficient = 0.0047, *P* = 0.88, **S2 Table**).

## Discussion

*In vitro* studies have identified A3F as one of the human APOBEC3 proteins with intrinsic anti-HIV-1 activity, and A3F unlike A3G is partially resistant to degradation by HIV-1 Vif [7,15–17]. In this population-based genetic study, we assessed the impact of *A3F* variation on HIV-1 disease progression in longitudinal HIV-1/AIDS natural disease cohorts. We found that the haplotype encoding *A3F* p. 231V and bearing 5 other variant alleles was significantly associated with slower progression to AIDS and lower viral load set-point. This protective effect is most pronounced for PCP, the most common AIDS-defining disease in the era before effective antiretroviral therapy was available. Our results provide genetic evidence supporting an active role of human A3F in modulating HIV-1 disease *in vivo*. The observed population level associations point toward the physiological relevance of A3F, which has long been debated [7,15–21].

A3F suppresses HIV-1 replication through deaminase-induced G-to-A hypermutations in viral DNA, as well as deamination-independent impairment of viral reverse transcription and prevention of HIV DNA integration [15–17,39,40]. The mechanism by which the *A3F* variant haplotype influences AIDS pathogenesis is not clear. Mechanistically, the expectation would be that A3F variant protein isoforms have different abilities in inhibiting HIV replication, either by change of protein function or abundance. The association of p.231V with lower viral load supports the idea that A3F ancestral protein isoform containing p. 231V is more effective in restricting HIV replication compared to the derived protein isoforms. In an *in vitro* HIV-1 infectivity experiment, comparable or slight stronger inhibitory activity against an HIV-1 X4 strain with the wild type Vif was observed for A3F 231V (45% infectivity) compared with A3F 231I (57% infectivity) [21]. It is possible that over time the inhibitory effects of the variant protein may lead to detectable differences in disease outcomes. In a HIV-1 vif-A3F degradation assay, we have also observed that A3F I231V can influence A3F sensitivity to certain HIV-1 Vif proteins. Altered Vif sensitivity of A3F variation may be a contributing factor for the observed differences in HIV-1 viral loads in our study population. Direct Sanger sequencing of HIV-1 viral RNA in plasma of patients in the Swiss HIV cohort identified very low levels of G to A hypermutation, which may represent a small subset of the variation present in the integrated proviral HIV. Although HIV G to A variation mediated by A3F was not associated with the *A3F* I231V genotype, deeper sequencing provirus may be needed to accurately access the role of *A3F* genetic variants on HIV editing [41,42]. Further studies are also needed to determine if *A3F* variants affects A3F protein function in blocking reverse transcription and or HIV integration.

Pneumocystis pneumonia, or PCP, caused by the fungus Pneumocystis jirovecii, is the most common opportunistic infection in untreated AIDS patients [43,44]. PCP often occurs in those with low CD4+ cell counts <200/mm^3^; in the MACS cohort, 30% of those patients developed PCP within 3 years after CD4+ cell counts dropping to <200/mm^3^ [43]. *A3F* variation impact on PCP may be related with altered activity of A3F in the pulmonary inflammatory environment after CD4+ cell decline. The pathogenesis of PCP is marked with the immune-mediated pulmonary inflammation involving chemokines and cytokines [44]. Inflammatory stimuli such as lipopolysaccharide and interferon-α potently induce A3G and A3F protein expression [45]. Research will be needed to determine if A3F protein is activated in lung inflammation sites, affecting local viral replication and immune response.

Our study is limited in sample size, especially as the individual cohorts represent different HIV risk factors and transmission routes; and we are underpowered in the African American group to detect small or modest effect sizes. In African Americans, lack of significant association could be due to small sample size and lower PCP rate (11%) compared to European Americans (19%). It is also possible that other operational SNPs in African Americans may be tracking or interacting with *A3F* 231V. Our results require further replication in other well-powered cohorts with well-defined clinical AIDS outcome data. The strong linkage disequilibrium among multiple SNPs within the *A3F* gene with various potential functional effects makes it difficult to clearly identify the causal variants responsible for the phenotypic associations. In addition to the p. 231V site, other variant alleles carrying on the haplotype may also potentially affect *A3F* gene regulatory function as suggested by differential DNA-protein binding conferred by the intronic SNP rs5750728. Although *A3F* SNPs were not in strong LD with other *A3* genes, the possibility of *A3F* SNPs tracking other SNPs in the *A3* gene family region cannot be completely ruled out due to the high homology among the gene family that pose technical difficulties in gene-specific genotyping, sequencing and assembly [46].

Our results may shed light on the relative contribution of A3 family members to HIV pathogenesis *in vivo*, a critical unanswered question. We and others have identified associations of a number of genetic variants in the *A3* family genes with HIV-1 acquisition or disease progression [28–30,33]. The H186R of *A3G* was associated with rapid progression to AIDS in African Americans [30], higher viral load in South African infected women [47] and rapid disease progression in infected children [48]. The C40693T variant of *A3G* [28] [33]may be associated with increased risk to acquisition, whereas Haplotype I of *A3H* may confer resistance to infection [29]. A3B has been shown to restrict HIV-1 in experiments using 293 cells lines [3,7,49–51], but not in the more biologically relevant T-cell lines [52,53]. The impact of the *A3B* gene deletion on HIV-1 infection and progression is inconsistent, possibly due to differences in study designs and case-control selection. We previously reported that the homozygous *A3B* deletion (D/D) was associated with increased risk to HIV-1 acquisition by comparing HIV-1 exposed but uninfected individuals to HIV-1 incident seroconverters, and further that seroconverters progressed more rapidly to clinical outcomes[33]. In support of this, one study found that the D/D carriers had a six-fold greater likelihood of having lower CD4+ T cell levels [54]. Other studies found no associations for infection or progression, but these were suffered from frailty bias which might have inflated type 2 errors. For example, one study used healthy controls and long-term non-progressors but not fast progressors [55,56], and the other study used a control group of HIV-1 negative MSM who were nearly a decade younger than infected cases[57]; given equal exposure time, many of the control group may have become infected. This study did show tendency to lower CD4+ T cell depletion with the *A3B* deletion during 88 day follow-up [57]. The role of A3B and the *A3B* deletion in HIV disease warrants further clarification as their role in multiple virus, pathogens and cancers increasingly has been recognized [54,58–61].

In this study, we identified a common haplotype tagged by *A3F* 231V variant as a novel AIDS-modifying genetic factor in European Americans, which shows a similar trend in African Americans with HIV infection. *A3G* H186R is only found in individuals with African ancestry and is almost absent in European population [30], while the *A3B* gene deletion is more common in individuals from Asia and is less frequent in Europeans, and is nearly absent in populations with West African ancestry. The differences in *APOBEC3* variant frequencies and haplotype structure among continental populations might have arisen from population-specific demographic events and or from viral selective pressure acting on the *APOBEC3* genes. This study adds new evidence that A3F plays an important role in restricting pathogenesis of HIV-1 in its natural host. These data support the development of therapeutics targeting the Vif-A3F axis, along with current efforts on A3G-Vif.

## Materials and Methods

### Study participants

We used clinically well-characterized subjects from 6 pretreatment HIV/AIDS cohorts. Study participants (N = 4203) were enrolled in USA-based, prospective natural history HIV/AIDS cohorts: Multicenter AIDS Cohort Study (MACS) [62], the San Francisco City Clinic Cohort Study (SFCCC) [63], AIDS Link to the Intravenous Experience (ALIVE) [64], Hemophilia Growth and Development Study (HGDS) [65], the Multicenter Hemophilia Cohort Study (MHCS) [66] and DC gay (DCG) [67]. These cohorts were initiated during 1980’s and participants were followed up semi-annually with blood draws for viral load and CD4+ T cell measurements and physical examinations at each visit. These treatment naïve cohorts have been used to identify multiple HIV-1/AIDS modifying genetic factors [30,68].

The study group includes 707 European American HIV-1 incident seroconverters, 281 African American incident seroconverters, 1135 HIV-uninfected at-risk individuals and 2076 HIV seroprevalent individuals who were HIV infected at study entry. The censoring date was the earliest of the date of the last recorded visit, or July 31, 1997 for the ALIVE cohort, or December 31, 1995 for all other cohorts, to avoid the confounding effect of highly active anti-retroviral therapy (HAART). A later censoring date was used for ALIVE cohort because few ALIVE participants received HAART prior to July 31, 1997 [69]. PCP prophylaxis status was only available for a subset of the MACS cohort; the first self-reported drugs used included Trimethoprim/sulfamethoxazole, aerosolized Pentamidine and Dapsone; the combination or switch-over of these drugs were used over the clinical course.

### Ethics statement

Ethical approval for the study was obtained from the National Institute of Health Office of Human Subjects Research Protections with OHSRP #3314. Ethical approval was obtained from institutional review boards for each of the respective contributing centers in the International Collaboration for the Genomics of HIV. Written informed consent was obtained from all study participants.

### Selection of *A3F* genetic variants

We selected six SNPs that were codon-changing, in a predicted splicing or regulatory site (based on SNPinfo web server [http://snpinfo.niehs.nih.gov]), considering SNP spacing and gene coverage. Several other nonsynonymous variants in A3F are reported in the 1000 genome project but were not genotyped here either because they were rare or tagged by other SNPs: rs2020390 (p. A108S) is in near-absolute LD (*r*^2^ = 0.94) with rs2076101 (p.I231V) in Europeans. rs34182094 (p. A178T), rs12157816 (p. Y307C), rs13056825 (p.N346S) were absent or infrequent in Utah Europeans (CEU) and Yoruba Africans (YRI).

### Genotyping of *A3F* genetic variants

SNPs were genotyped using the TaqMan allele discrimination assays on an ABI 7900HT sequencer detector system (Applied Biosystems), according to the manufacturer’s protocol. For quality control, water controls were included on each plate and 10% of samples were duplicated. No water contamination or genotype mismatches between duplicates was observed. SNP rs2076101 was also genotyped by a second in-house designed TaqMan assay (sequences available upon request); the resulted genotypes were identical by two assays.

### Electrophoretic mobility shift assays (EMSA)

EMSA was performed in nuclear extracts from HeLa cell stimulated with PMA using oligonucleotides carrying *A3F* rs5750728 T/C with an Infrared EMSA Kit (LI-COR), as previously described [70]. The double-stranded oligonucleotides used were (SNP allele underlined): 5’-CCCTTTGGCCAGTGCGTCCCACCACAT-3’ and 5’-CCCTTTGGCCAGTGCGCCCCACCACAT-3’.

### Cell culture, transfection and immunoblot analysis

HEK293T (ATCC, catalog no. CRL-11268) cells were maintained in Dulbecco's modified Eagle's medium (DMEM, Invitrogen) with 10% fetal bovine serum and penicillin/streptomycin (D-10 medium) and passaged upon confluence. DNA transfection in HEK293T cells was carried out using Lipofectamine 2000 (Invitrogen) as recommended by the manufacturer. For degradation assays, HEK293T cells in 12-well plates were transfected with 1 μg of Vif or 1 μg of empty vector and 0.3 μg of A3F expression vector at a 3:1 ratio. HEK293T cells were collected at 48 h after transfection. Cell lysates were lysed in 1x loading buffer (0.08 M Tris, pH 6.8, with 2.0% SDS, 10% glycerol, 0.1 M dithiothreitol and 0.2% bromophenol blue) and boiled for 5 min before separating the proteins by SDS-PAGE. Membranes were probed with various primary antibodies against proteins of interest. Secondary antibodies were alkaline phosphatase-conjugated anti-sheep, anti-rabbit or anti-mouse (Jackson Immunoresearch) antibodies, and staining was carried out with 5-bromo-4-chloro-3-indolyl phosphate (BCIP) and nitroblue tetrazolium (NBT) solutions prepared from chemicals obtained from Sigma.

### Detection of HIV-1 hypermutation

HIV-1 sequences covering the *pol* gene alone (in half of the patients) or the *pol* gene with additional other genes were obtained by the Sanger sequencing from plasma of patients in the Swiss HIV Cohort Study. A3F specific hypermutation was quantified as in the Los Alamos Hypermut2 tool [38]. Consensus nucleotide sequences of the samples and of the hxb2 reference sequence were translated to amino acid sequence from which a multiple alignment was created with muscle [71]. Using the amino acid sequence alignment we derived a codon-aware nucleotide alignment, which was used to count the number of possible hypermutation sites. We counted the number of trinucleotides matching the “GA[A or T or G]” pattern in the reference sequence as the number of potential A3F hypermutation sites. The number of actual A3F induced mutations in each sample was determined as a subset of potential A3F hypermutation sites where the first nucleotide was an A. It should be noted that GA → AA editing can be mediated by A3D/F/H [39,41]. To quantify the background mutation level we used the following regular expression, which is the same as in the Hypermut2 tool: “G(([CT][ACGT])|([AG]C))”. The level of hypermutation of a patient sample was determined as an odds ratio: (number of A3F induced mutations/number of potential A3F specific sites) / (number of background mutations / number of potential background sites). Statistical tests were carried out on the logarithm of the latter quantity using linear regression. The regression included host PCA axes as covariates, the allele dosage of rs2076101, and an indicator variable indicating the SNP genotyping platform.

### Statistical analysis

We performed analyses using SAS version 9.12 (SAS Institute, Cary, NC).

We assessed the influences of the *A3F* variants on disease progression to AIDS outcomes by the Cox proportional hazards model (Cox model) and Kaplan-Meier survival curve analyses. We only included seroconverters with known infection dates (midpoint estimation between last seronegative and first seropositive HIV test) in our analysis. The endpoint was clinical AIDS diagnosis using the Centers for Disease Control and Prevention (CDC) 1987 definition of AIDS [34], i.e. HIV-1 infection plus AIDS-defining illness or AIDS-related death. The median time from seroconversion to AIDS was 10 years in European American seroconverters. As the Kaplan-Meier survival analysis indicated that the haplotype tagged by rs2076101 best fitted a dominant genetic model, we compared the heterozygous (231I/V) and homozygous genotype (231V/V) state to the reference group of homozygotes (231I/I) in a Cox proportional hazards model. European American and African American groups were analyzed separately because the allele frequencies were different between the two groups. We included known genetic factors modifying AIDS progression as covariates in the adjusted Cox model analysis: *CCR5* Δ32, *CCR5*-59029 (*CCR5*-2459, rs1799987), *HLA*-B*27, *HLA*-B*57, *HLA*-B*35Px group (including *HLA*-B*3502, B*3503, B*3504, and B*5301), *HLA-C* [32] and *HLA* Class I homozygosity for European American (reviewed in [72]; *HLA*-B*57 and *HLA* Class I homozygosity for African American. We stratified the analyses by sex and by age at seroconversion: 0–20, >20–40, and > 40 years. P values for Cox model analysis were from the Wald test. Although our previous GWAS for association with HIV phenotypes in EA indicated minimal population substructure (genomic inflation factor λ = 1.01), we conservatively include the first two eigenvalues from a PCA analysis (EigenSoft) to correct for population stratification [73].

HIV-1 viral load set-point was defined as the mean log_10_-transfromed copies of HIV-1 RNA in plasma measured between months 6–33 after seroconversion (2–5 measurements). Viral load measurements exceeding three-fold (0.5 log_10_) from the average of all remaining points were excluded, as previously suggested [35]. We used the *t* test for analysis of differences between viral load means of the group carrying 0 or 1 231V allele versus the 231I homozygote group.

The International Collaboration for the Genomics of HIV (ICGH) combined the GWAS data from 25 cohorts from Europe and North America, including cohorts used in this study [36]. The genotypes of the *A3F* SNPs from ICGH were obtained from the previous GWAS data [36]. The impact of the *A3F* SNPs on HIV-1 viral load in ICGH was assessed using a fixed-effect inverse-variance weighted meta-analysis as previously described [36]. A summary of the selected study groups and cohorts used in the meta-analysis is presented in **S3 Table**. Viral load data from a total of 10395 (7266 European Americans, 3129 African Americans) HIV-1 seropositives was used in the meta-analysis.

The impact of *A3F* variants on HIV-1 infection susceptibility was assessed by comparing allelic frequencies between the HIV-1 infected group comprising seroconverter and seroprevalent persons and the HIV-1 uninfected group composed of persons at risk for HIV. Odds ratios (OR) and *P* values were obtained by using a conditional logistic regression test. All *P* values were 2-tailed.

## Supporting Information

S1 Table Distribution of *A3F*-231I/V in the HIV-1 negative and positive groups. (DOCX)

S2 Table HIV-1 hypermutations detected from patients in the Swiss HIV Cohort Study, stratified by the *A3F*- rs2076101 genotypes. (DOCX)

S3 Table Summary of cohorts in the International Collaboration for the Genomics of HIV (ICGH) consortium used for HIV-1 viral load analysis. (DOCX)

S1 Fig Linkage disequilibrium in the *APOBEC3* gene region. Data was based on HapMap phase III data in the CEU (Utah Caucasian) population and was plotted with Haploview. The intensity of the box reflects the r^2^ level and haplotype block was defined by 95% CI.
(TIFF)

S2 Fig Frequency distribution of *A3F*-231I/V genotypes in European Americans of fast progressors and slow progressors. (TIFF)

S3 Fig Kaplan-Meier survival curves of *A3F*-231I/V genotype carriers for progression to PCP since HIV-1 infection in 278 African American seroconverters. RH and adjusted *P* values were obtained from the Cox proportional hazards model. *P* values for survival curves were obtained from the log-rank test.
(TIFF)
