# Supplementary material for: Role of APOBEC3F Gene Variation in HIV-1 Disease Progression and Pneumocystis Pneumonia
Source: PLoS Genet. 2016 Mar 4;12(3):e1005921. doi: 10.1371/journal.pgen.1005921 (PMC4778847; doi:10.1371/journal.pgen.1005921)
Supplement: S1 Table — (DOCX) [file pgen.1005921.s001.docx]

| **S1 Table. Distribution of *A3F* 231V in the HIV-1 negative and positive groups** | | | | | |
| --- | --- | --- | --- | --- | --- |
| **Population** | **HIV-1 status** | **n** | **allele freq. of A3F 231V** | **OR (95% CI)*** | |
| EA | HIV-1 - | 694 | 0.46 | - | |
|  | HIV-1 + | 2028 | 0.49 | 1.08 (0.89, 1.31) | |
| AA | HIV-1 - | 440 | 0.74 | - | |
|  | HIV-1 + | 1036 | 0.76 | 1.03 (0.66, 1.63) | |
| * chi square test for a dominant model. | | | | |  |
