# Supplementary material for: Role of APOBEC3F Gene Variation in HIV-1 Disease Progression and Pneumocystis Pneumonia
Source: PLoS Genet. 2016 Mar 4;12(3):e1005921. doi: 10.1371/journal.pgen.1005921 (PMC4778847; doi:10.1371/journal.pgen.1005921)
Supplement: S2 Table — (DOCX) [file pgen.1005921.s002.docx]

**S2 Table. HIV-1 hypermutations detected from patients in the Swiss HIV Cohort Study,**

**stratified by the rs2076101 genotypes**

| Allele dosage of rs2076101 | | 0 | 1 | 2 | |
| --- | --- | --- | --- | --- | --- |
| Number of Apobec3F specific G->A mutations (A) | | 8.11 | 8.23 | | 8.63 |
| Number of potential Apobec3F specific G->A mutations (B) | | 144.95 | 147.90 | | 163.72 |
| Number of background G->A mutations (C) | | 14.57 | 14.77 | | 15.56 |
| Number of potential G-> A mutations (D) | | 225.35 | 228.87 | | 255.69 |
| Odds ratio: ln( (A/B)/(C/D) ) | | -0.15 | -0.08 | | -0.15 |
|  | Coefficient* | | Std. Error | | *P*-value |
| (Intercept) | -0.11 | | 0.04 | | 0.009 |
| rs2076101 (allele dosage) | 0.0047 | | 0.03 | | 0.88 |

* Additive model from linear regression of In OR with allele dosage.
