# Supplementary material for: Role of APOBEC3F Gene Variation in HIV-1 Disease Progression and Pneumocystis Pneumonia
Source: PLoS Genet. 2016 Mar 4;12(3):e1005921. doi: 10.1371/journal.pgen.1005921 (PMC4778847; doi:10.1371/journal.pgen.1005921)
Supplement: S3 Table — (DOCX) [file pgen.1005921.s003.docx]

| **S3 Table. Summary of cohorts in the International Collaboration for the Genomics of HIV (ICGH) consortium used for HIV-1 viral load analysis** | | | | |  |
| --- | --- | --- | --- | --- | --- |
| **Cohort**  **code** | **N total** | **Country(ies)** | **Ethnicity** | **Center(s), cohorts** | **GWAS Platform(s)** |
| acs_eur | 408 | Amsterdam | European | Amsterdam Cohort Study | Illumina 300 |
| cs1_aam | 380 | USA | African American | International HIV Controllers Study/AIDS Clinical Trials Group | Illumina 650 |
| cs1_eur | 505 | USA | European | International HIV Controllers Study/AIDS Clinical Trials Group | Illumina 650 |
| cs2_aam | 379 | USA | African American | International HIV Controllers Study/AIDS Clinical Trials Group | Illumina 1M |
| cs2_eur | 578 | USA | European | International HIV Controllers Study/AIDS Clinical Trials Group | Illumina 1M |
| cs3_aam | 412 | USA | African American | International HIV Controllers Study/AIDS Clinical Trials Group | Illumina 1M |
| cs3_eur | 580 | USA | European | International HIV Controllers Study/AIDS Clinical Trials Group | Illumina 1M |
| ecs_eur | 1507 | Switzerland, Spain, Italy and other European | European | European Center for HIV Vaccine Immunology (EuroCHAVI)/Swiss HIV Cohort Study | Illumina 650 |
| fgp_eur | 962 | France | European | GRIV, PRIMO | Illumina 300 |
| lgd_aam | 850 | USA | African American | Laboratory of Genomic Diversity (NIH), ALIVE, DCG, HGDS, MHCS, SFCC | Affymetrix 6.0 |
| lgd_eur | 1476 | USA | European | Laboratory of Genomic Diversity (NIH), ALIVE, DCG, HGDS, MHCS, SFCC | Affymetrix 6.0 |
| md1_eur | 743 | USA | European | Duke University, Multicenter AIDS Cohort Study (MACS) | Illumina 1M |
| md2_eur | 268 | USA | European | Duke University, MACS | Illumina 550 |
| mdo_aam | 578 | USA | African American | Department of Defense, MACS | Illumina 1M |
| uhs_aam | 530 | USA | African American | Research Triangle, Urban Health Study (UHS) | Illumina 1M |
| uhs_eur | 239 | USA | European | Research Triangle, UHS | Illumina 1M |

- European (eur) and African American (aam) participants in this table contributed to the results in Fig. 4A and 4B, respectively.
