# Supplementary figures and images for: Role of APOBEC3F Gene Variation in HIV-1 Disease Progression and Pneumocystis Pneumonia
Source: PLoS Genet. 2016 Mar 4;12(3):e1005921. doi: 10.1371/journal.pgen.1005921 (PMC4778847; doi:10.1371/journal.pgen.1005921)

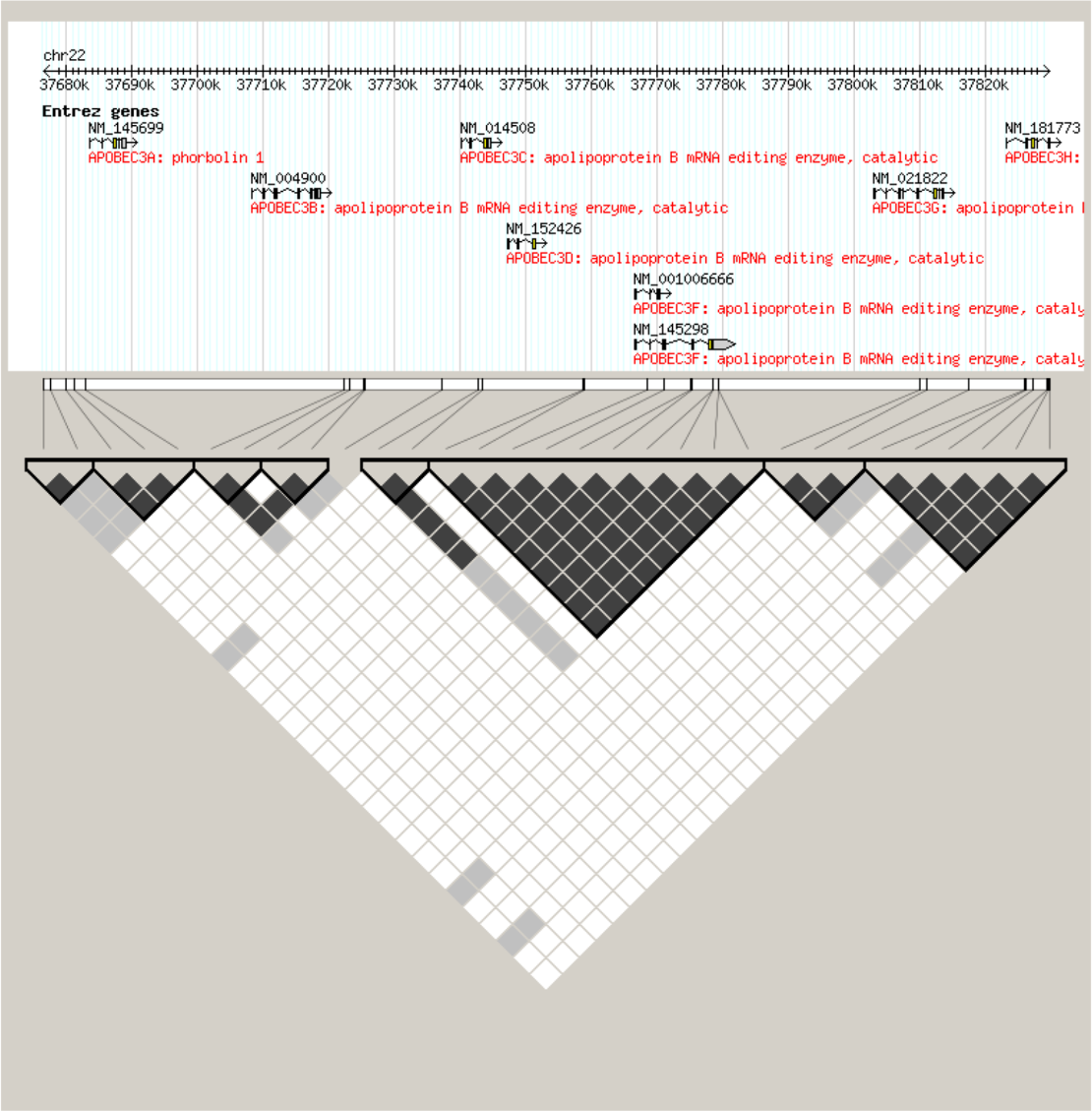

Supplement: S1 Fig — Data was based on HapMap phase III data in the CEU (Utah Caucasian) population and was plotted with Haploview. The intensity of the box reflects the r2 level and haplotype block was defined by 95% CI. (TIFF) [file pgen.1005921.s004.tiff]

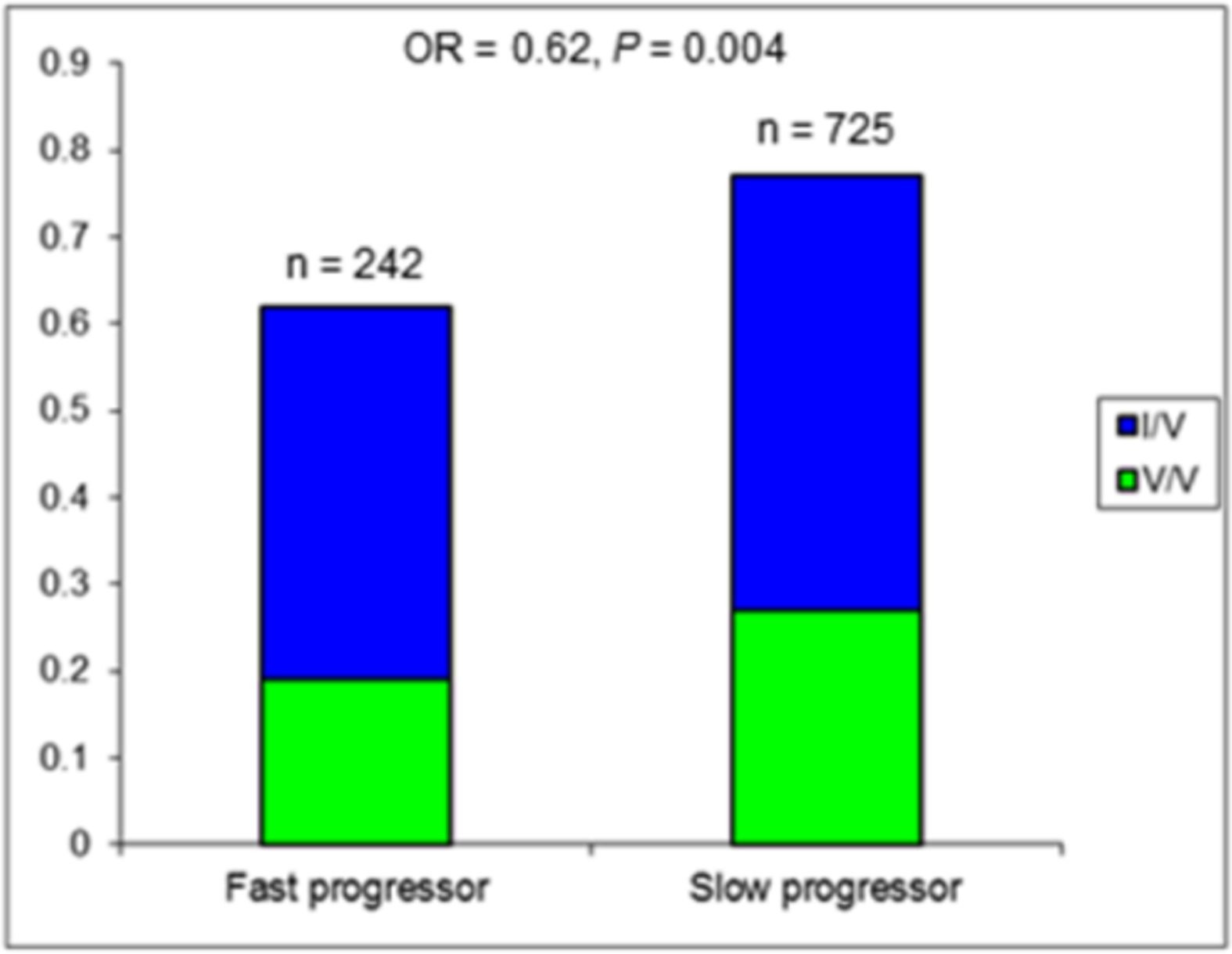

Supplement: S2 Fig — (TIFF) [file pgen.1005921.s005.tiff]

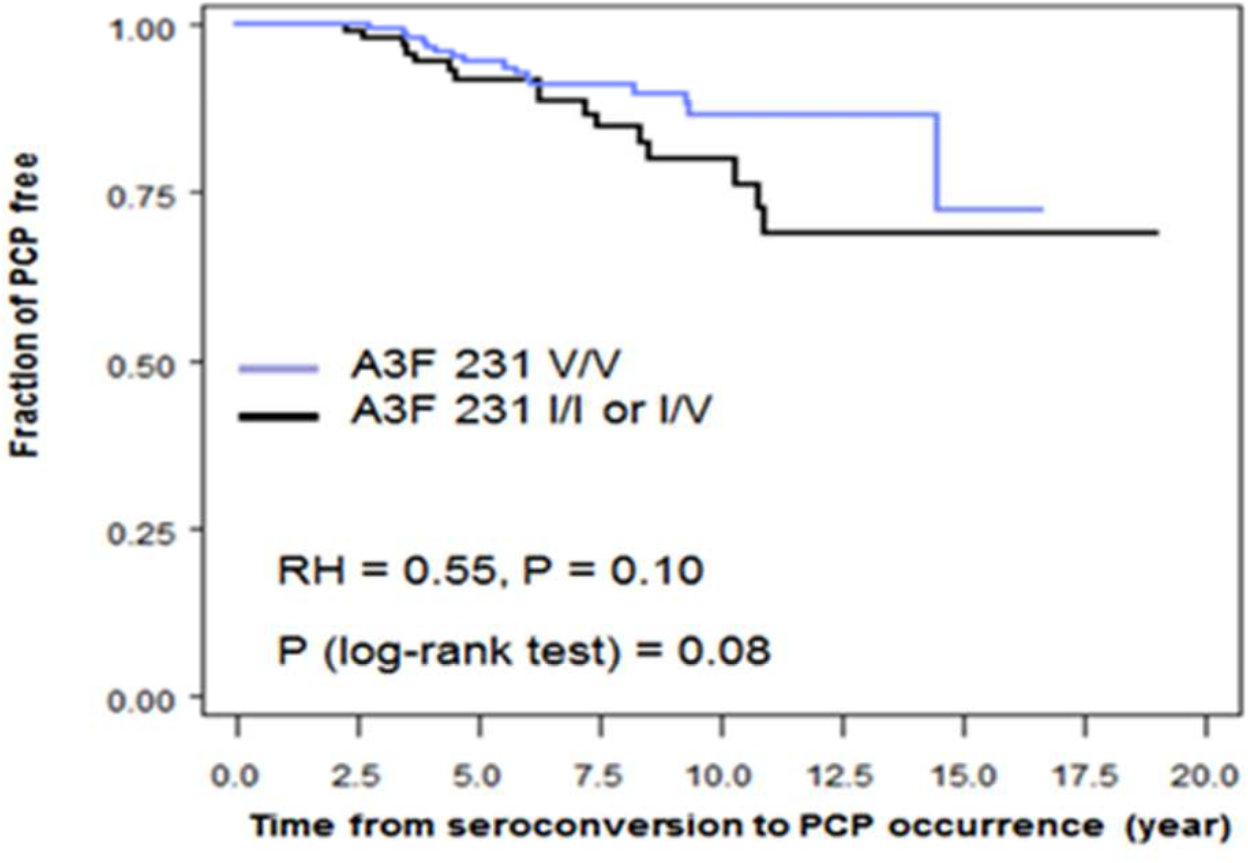

Supplement: S3 Fig — RH and adjusted P values were obtained from the Cox proportional hazards model. P values for survival curves were obtained from the log-rank test. (TIFF) [file pgen.1005921.s006.tiff]
